# Rassf Proteins as Modulators of Mst1 Kinase Activity

**DOI:** 10.1038/srep45020

**Published:** 2017-03-22

**Authors:** Aruna Bitra, Srinivas Sistla, Jessy Mariam, Harshada Malvi, Ruchi Anand

**Affiliations:** 1 Department of Chemistry, Indian Institute of Technology Bombay, Powai, Mumbai, 400076, India; 2 GE Healthcare Life Sciences, John F Welch Technology Centre, Whitefield Road, Bangalore, 560048, India

## Abstract

Rassf1A/5 tumor suppressors serve as adaptor proteins possessing a modular architecture with the C-terminal consisting of a coiled-coil SARAH (Salvador-Rassf-Hippo) domain and the central portion being composed of Ras associated (RA) domain. Here, we investigate the effect of Rassf effectors on Mst1 function by mapping the interaction of various domains of Rassf1A/5 and Mst1 kinase using surface plasmon resonance (SPR). The results revealed that apart from the C-terminal SARAH domain of Mst1 which interacts to form heterodimers with Rassf1A/5, the N-terminal kinase domain of Mst1 plays a crucial role in the stabilization of this complex. In addition, SPR experiments show that the RA domains play an important role in fine-tuning the Mst1-Rassf interaction, with Rassf5 being a preferred partner over a similar Rassf1A construct. It was also demonstrated that the activity profile of Mst1 in presence of Rassf adaptors completely switches. A Rassf-Mst1 complexed version of the kinase becomes apoptotic by positively regulating Mst1-H2B mediated serine 14 histone H2B phosphorylation, a hallmark of chromatin condensation. In contrast, the heterodimerization of Mst1 with Rassf1A/5 suppresses the phosphorylation of FoxO, thereby inhibiting the downstream Mst1-FoxO signalling pathway.

Rassf family of tumor suppressors are novel class of adaptor proteins that primarily mediate their function by initiating a cascade of protein-protein interactions^1^,^2^,^3^. These proteins interact with the activated GTP-Ras form via their RalGDS/Af6 Ras associated (RA) domain^4^, a mechanism that enables sequestering of any misregulated activated Ras pool. There are multiple Rassf isoforms among which Rassf1A, Rassf2 and Rassf5 are the most well characterized members^5^,^6^,^7^,^8^. Apart from the Ras binding RA domain, both Rassf1A and Rassf5 contain another coiled-coil adaptor domain at the extreme C-terminal end, known as Salvador-Rassf-Hippo (SARAH)^1^. The SARAH domain, is a common motif that is shared by several proteins and is responsible for promotion of protein-protein interactions among the members possessing it^9^. It is via this SARAH domain that Rassf set of effectors, complex with another protein Mst1 kinase (mammalian sterile 20-like kinase 1)^10^.

Mst1 is a pro-apoptotic kinase and its overexpression induces apoptosis in a variety of cellular backgrounds that involve activation of stress related pathways^11^. It belongs to the serine/threonine family of sterile 20 (Ste20) proteins^12^,^13^ and exists in two forms; a 54 kDa full length protein and a 36 kDa caspase cleaved version^14^. The truncated version of Mst1 only consists of the kinase domain, while the full length enzyme contains an additional C-terminal regulatory region along with the homodimerization SARAH domain^15^,^16^. The two established substrates of Mst1 are the nucleosomal component protein, histone H2B and the transcription factor forkhead box O (FoxO)^17^. In 2008, Marmorstein and co-workers demonstrated that various isoforms of Mst1 show differential kinase activity towards its substrates^17^. As opposed to histone H2B, the Mst1-fl has a 16 fold higher catalytic efficiency towards FoxO, with the C-terminal regulatory domain being crucial for catalyzing the phosphorylation^17^. The Mst1-N kinase enzyme that is devoid of C-terminal regulatory and homodimerization domains, exhibits 30 fold higher *k*_cat_/*K*_M_ for phosphorylation of histone H2B. In 2003, Allis and co-workers showed that the truncated caspase cleaved form of Mst1 which phosphorylates histone H2B^18^ leads to chromatin condensation, DNA fragmentation and ultimately cell death, promoting apoptosis^19^. The full length cytosolic version of Mst1 phosporylates FoxO at a conserved serine residue (Ser 207 in FoxO3 and FoxO1 at Ser 212)^20^ within the winged helix DNA binding domain^21^,^22^. In addition, in this region of FoxO, a variety of other posttranslational modifications also occur that in consort mediate its transcriptional activity^23^. In particular, the Mst1 mediated FoxO signalling has been shown to induce activation of FoxO via disruption of its complex with 14-3-3 leading to its subsequent migration to the nucleus^24^. This activation event leads to cell death in mammalian neuronal cells^20^,^21^. In nematodes, the Mst1-FoxO signalling is implicated in promoting life span via delay in tissue aging^21^.

The interaction between Ras and the RA domain of Rassf5 is structurally characterized and it has been demonstrated that the complex is very stable and has an extended contact area^25^. Furthermore, various biochemical studies corroborate these findings and RA domain of Rassf5 and Ras has been shown to form a complex possessing a long lifetime (~10 s) with the *K*_D_ value being in the nanomolar range^6^,^25^. It appears that all Rassf isoforms do not exhibit the same affinity for Ras-GTP. For example, the interaction of Rassf1A with Ras is thirty three times weaker than the Rassf5-Ras complex^25^. This highlights that *in vivo* Rassfs may serve as adaptors in multiple signalling pathways and may not be a redundant set that possess the same physiological partners. Apart from the RA domain interactions of Rassf proteins, the SARAH domain interactions have also been structurally characterized. X-ray structure of the SARAH-SARAH coil-coil domains of Mst1 and Rassf revealed that they both interact to form heterodimers. The complex consists of helical antiparallel homodimers/heterodimers with the hydrophobic residues stabilizing the interface, a common mode used by this fold to mediate protein-protein contacts^6^,^26^,^27^,^28^,^29^.

Even though there is a lot of literature available on Rassf interactions there are several aspects of the interaction of Rassf with Mst1 which remain elusive. For example, it is not clear whether the various Rassf isoforms like Rassf1A and Rassf5 form unique Mst1-Rassf complexes and exhibit differential affinity or whether this interaction is generic in nature. It is highly likely that other domains of Rassf and Mst proteins also play an important role in fine-tuning the specificity of this interaction. The other pressing question pertains to understanding the effect of the interaction of Rassf with Mst1 towards its physiological substrates. This is important as it emphasizes the role played by protein-protein interactions in regulating substrate scope, thereby altering downstream signalling that control the fate of the cell. Finally, since Rassf proteins have the ability to independently dock both Ras-GTP and Mst1 kinase, it would be interesting to explore if Ras-GTP and Mst1 use Rassf as an adaptor platform to communicate. In the present work, the above mentioned aspects of Rassf interaction on Mst1 function have been explored by employing a combination of surface plasmon resonance (SPR) and kinase assay experiments. Together the information obtained was employed to develop a mechanistic model detailing the Rassf mediated Mst1 activity.

## Results and Discussion

### Binding studies of Mst1 with Rassf1A and Rassf5A

**Figure 1:**
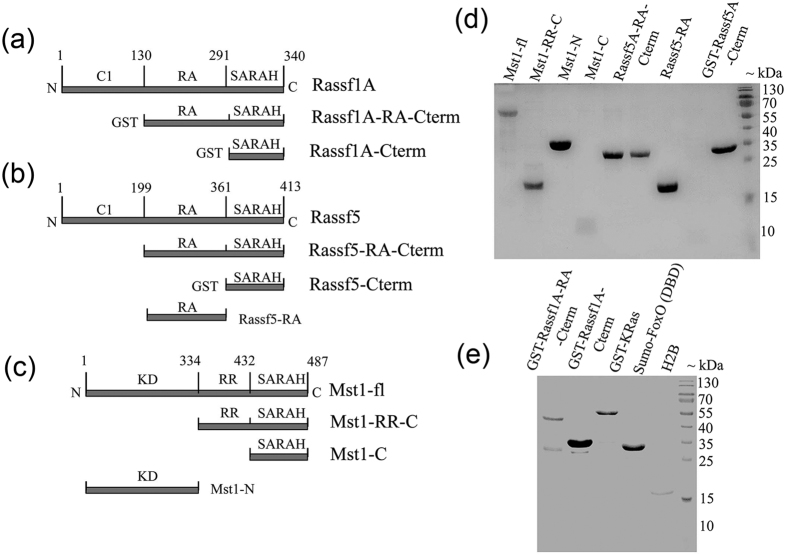
Domain organization of Rassf family proteins and Mst1. All the construct designs for (**a**) Rassf1A (**b**) Rassf5A and (**c**) Mst1 used for this study are depicted here. (**d**) and (**e**) SDS-PAGE gel depicting the purity of various protein constructs used in this study. Different domains are abbreviated as follows: SARAH, Salvador-Rassf-hippo domain; C1, N-terminal C1 type zinc fingers; RA, Ras binding domain; KD, kinase domain; RR, regulatory region. Various constructs are abbreviated as follows: Rassf1A-RA-Cterm, Rassf1A protein containing Ras binding domain and SARAH domain (130–340); Rassf1A-Cterm, only SARAH domain (291–340); Rassf5A-RA-Cterm, Rassf5A protein containing Ras binding domain and SARAH domain (200–413); Rassf5A-Cterm, only SARAH domain (361–413); Rassf5A-RA, only Ras binding domain (200–365); Mst1-fl, full length Mst1 kinase; Mst1-RR-C, Mst1 kinase containing regulatory region and C-terminal SARAH domain (354–487); Mst1-C, only SARAH domain (432–487).

Determination of both the specificity and strength of binding interaction is paramount towards deciphering the underlying mechanism of action. By employing SPR as a primary tool we have interpreted the effect of different regions of both Mst1 and Rassf1A/5A proteins towards their SARAH-SARAH domain association. Variants of Rassf1A, Rassf5A containing the C-terminal SARAH domain and Ras binding RA domain were constructed as shown in Fig. 1a and b. The purity of all the constructs was checked by SDS-PAGE (Fig. 1d and e). Circular dichroism studies were performed to ensure that the purified proteins were properly folded (Supplementary Figure S1). Modified Mst1 constructs, consisting of full length, the kinase domain deleted version with only the regulatory loop along with the SARAH domain and one containing only the extreme C-terminal SARAH domain (Fig. 1c) were subject to BIAcore experiments. In all studies the Rassf family proteins were used as ligands and the Mst1 constructs were used as analytes. The experimental values of all sensorgrams obtained from the SPR binding kinetics between different constructs of Rassf1A/5A and Mst1 were fitted using a 1:1 binding model and further analysis to obtain affinity and rate constants were performed.

### SPR kinetic analysis for interaction of Rassf1A/5A-SARAH and Rassf1A/5A-RA-SARAH domain with Mst1

**Figure 2:**
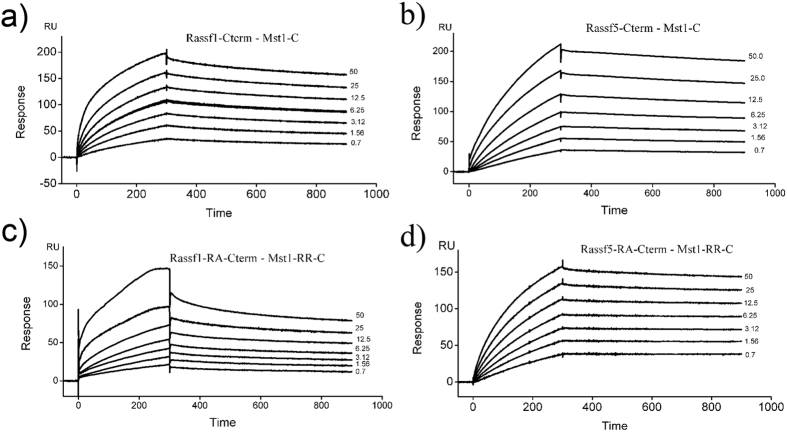
BIAcore analysis for the interaction of Mst1-C and Mst1-RR-C with Rassf1A/5A-C and Rassf1A/5A-RA-C surfaces. Sensorgrams are presented for the interaction of Mst1-C with (**a**) Rassf1A-Cterm (**b**) Rassf5A-Cterm; interaction of Mst1-RR-C with (**c**) Rassf1A-RA-Cterm (**d**) Rassf5A-RA-Cterm respectively. concentration range (50, 25, 12.5, 6.25, 3.125 and 0.7 μM) of Mst1 analytes was analyzed on Rassf1A/5A-C and Rassf1A/5A-RA-C biosensor surfaces at a flow rate of 30–45 μl/min in HBS running buffer and at temperature of 25 °C. The spike that appeared due to the bulk contribution (RI) was removed during final data fitting by adjusting injection events in the BIAcore T200 evaluation software.

The first set of SPR experiments were performed with the minimal constructs consisting of only the SARAH domains of both Rassf1A (Rassf1A-Cterm) and Rassf5A (Rassf5A-Cterm) with that of Mst1 (Mst1-C). The sensorgrams for the binding of Mst1-C with both Rassf1A-Cterm and Rassf5A-Cterm are shown in the Fig. 2a and b. Since the rate of dissociation of Mst1-Rassf complex is very slow, the kinetics for this interaction was performed by regenerating the sensor surface and freshly capturing the ligand for binding to each concentration of analyte. The kinetic analysis of the sensorgrams reveals that Mst1-C binds to both Rassf1A-Cterm and Rassf5A-Cterm with moderate *K*_D_ values in the high nanomolar range. This demonstrates that a strong interaction exists between the C-terminal SARAH domains of both Mst1 and Rassf set of proteins. To investigate whether regulatory region (RR) of Mst1 intervenes with the formation of Mst1-C – Rassf1A/5A-C complex, SPR experiments with longer constructs of Mst1 that comprise of the regulatory region in addition to the SARAH domain were performed (Mst1-RR-C). The kinetic analysis depicts slower association and faster dissociation rates for Mst1-RR-C with both Rassf1A-Cterm and Rassf5A-Cterm as compared to what is observed for Mst1-C complexes (Supplementary Figure S2 and Table 1). The results show that the Mst1-RR-C interaction with the SARAH domains of Rassf proteins is around 3 fold weaker than that of the construct containing only the SARAH domain of Mst1. We can therefore, conclude that addition of the regulatory domain partially destabilizes the Mst1-SARAH – Rassf-SARAH complex. The data also reveals that Mst1-C has a slight preference for binding to the Rassf5A over Rassf1A SARAH domain. A inspection of the sequences of the Rassf proteins depicts that the SARAH domains of both Rassf1A and Rassf5A share 51% sequence identity (out of 53 amino acids of Rassf5A-Cterm and 51 amino acids of Rassf1A-Cterm) and most of the amino acids responsible for interaction with Mst1 are conserved (Supplementary Figure S3). It is therefore not surprising that both exhibit almost similar order of magnitude in binding to Mst1 SARAH domain. However, a closer look at the sequences revealed that some interacting residues are different between the two sequences, for example, Glu380 in Rassf5A is replaced by Gln309 in Rassf1A and Phe397 is replaced by a cysteine residue. The recent crystal structure of the human Mst1/2-SARAH – Rassf5A-SARAH complex shows that both these residues are involved in stabilizing the heterodimeric SARAH-SARAH interface^28^,^29^,^30^. It is possible that these fine changes are responsible for differential recognition of the adaptors and lead to marginal preference of Mst1 towards Rassf5A as opposed to Rassf1A.

### SPR kinetic analysis for interaction of Rassf1A/5A-RA-SARAH region with Mst1

The tumor suppressor proteins Rassf1A and Rassf5A, as mentioned earlier also consist of a RA domain, located N-terminal and adjacent to the SARAH domain^1^. The RA domain of Rassf proteins, like other Ras effectors possesses an ubiquitin like fold that interacts with both the switch I and switch II regions of Ras^25^. To investigate whether RA domain plays a role in the overall binding of Rassf proteins to Mst1, SPR studies were undertaken. The Rassf1A-RA-Cterm and Rassf5A-RA-Cterm constructs were used as ligands and Mst1-C and Mst1-RR-C constructs as analytes (Fig. 2c,d and Supplementary Figure S2). The SPR data (Table 1) shows that addition of RA domain of Rassf1A and Rassf5A has a differential effect on each of these isoforms. In the case of Rassf1A, the presence of the RA domain decreases its binding with Mst1. While in Rassf5A, presence of RA domain did not interfere with SARAH heterodimerization. This is apparent from a ~10 fold decrease in binding affinity with Rassf1A in comparison to Rassf5A (Table 1). The data also shows that RA domain of Rassf1A and RR domain of Mst1 do not exhibit appreciable interaction. However, SPR experiments with RA domain of Rassf5A show that it exclusively interacts with the RR domain of Mst1 (Supplementary Figure S2 and Table 1). It is possible that this binding effect observed maybe due to other reasons like protein conformational changes, presence of extra sequences, aggregation etc. However, a plausible explanation could also be that the RA domain of Rassf5A is indeed designed to interact with the RR domain of Mst1 and assists it from deterring the SARAH-SARAH interactions. These findings seem to indicate that there exists selectivity of binding among the Rassf effectors mediated by their RA domains. A similar pattern of differential binding has also been previously reported between Rassf5A and Rassf1A binding towards GTP-Ras^25^. It appears while the SARAH domain end is a generic adaptor, the RA domain seems to provide fine-tuning among Rassf proteins, directing specificity of function.

### SPR studies with Mst1-fl and Rassf5A

**Figure 3:**
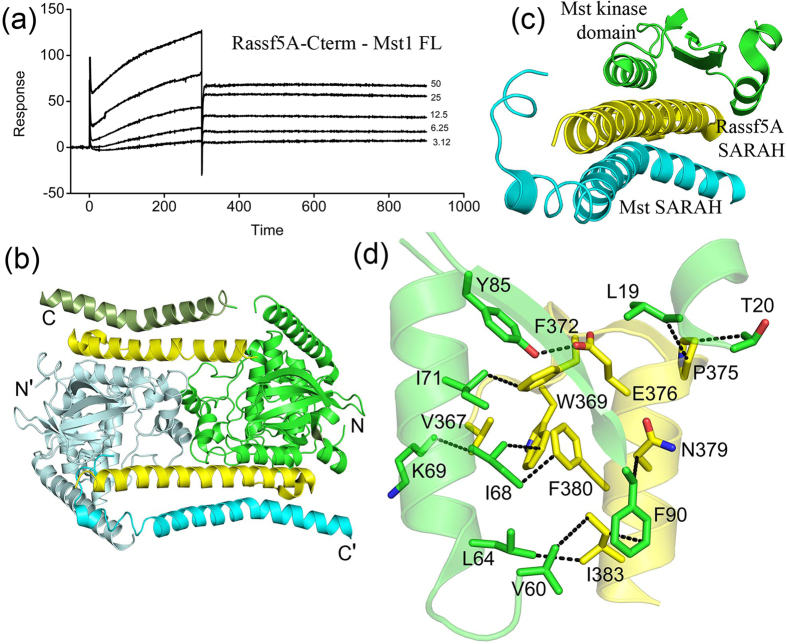
Interaction of Rassf5A-SARAH domain with Mst1 kinase. (**a**) BIAcore sensorgram depicting the interaction of Rassf5A-SARAH domain with full length construct of Mst1kinase. (**b**) Cartoon representation of crystallographic dimer of Mst2 (kinase domain and SARAH domain) – Rassf5A-SARAH complex. In monomer A, Mst2 kinase domain is colored green, SARAH domain in pale green; in monomer B, Mst2 kinase domain in pale cyan and its SARAH domain is in cyan. The Rassf5A-SARAH domain is indicated in yellow color. (**c**) Zoomed view of Mst2 – Rassf5A-SARAH complex depicting Rassf5A SARAH (yellow color) sandwiched between Mst2 kinase domain of monomer A (green color) and SARAH domain of monomer B (cyan color). (**d**) Interaction network between N-terminal kinase domain of Mst2 and N-terminal region of Rassf5A-SARAH domain. Carbon atoms of the Mst2 kinase domain are shown in green and that of the Rassf5A-SARAH domain in yellow, oxygen atoms in red color and nitrogen atoms in blue. All the figures were made in PyMOL using PDB code 4LGD.

To understand whether the N-terminal kinase domain of Mst1 has any effect on binding to Rassf adaptors, SPR studies using full length Mst1(Mst1-fl) as analyte were performed. The results show that the Mst1-fl − Rassf5A-Cterm complex is very stable and increasing the dissociation time further does not change the *k*_d_ value. The data indicates that Mst1-fl exhibits very strong binding affinity towards Rassf5A surface with an estimated *K*_D_ value in the low nanomolar range (Fig. 3a). The binding affinity of Mst1-fl is approximately 10 fold greater than the SARAH domain containing Mst1-C construct. This result was perplexing as we expected a sharp decrease in affinity due to the addition of a bulkier kinase domain. An analysis of the recently available crystal structure of Mst2 (homologue of Mst1) in complex with the Rassf5A C-terminal SARAH domain by Lisheng *et al*.^29^ was extremely helpful in explaining the SPR result. The structure clearly shows that the Rassf5A SARAH domain interacts with both the SARAH and kinase domains of Mst2 (Fig. 3b and c). The N-terminus helical region of Rassf5A SARAH domain packs with the extreme N-terminus helix of the Mst2 kinase domain resulting in ten hydrophobic interactions (Fig. 3d). There is also a hydrogen bonding interaction between the conserved Tyr85 of Mst2 with the Glu376 of Rassf5A. The remaining C-terminal extended portion interacts with the SARAH domain of Mst2 and as a result further anchors it. The structure shows that the SARAH domain of Rassf5A is sandwiched between the N-terminus kinase domain and the C-terminal SARAH domain of Mst2 (Fig. 3b and c). Because of this dual mode of interaction of the Rassf5A SARAH domain with distal regions of Mst, a dramatic increase in affinity is observed in the SPR experiments with Mst1-fl.

### Effect of Rassf1A/5 A proteins on the activity of Mst1

**Figure 4:**
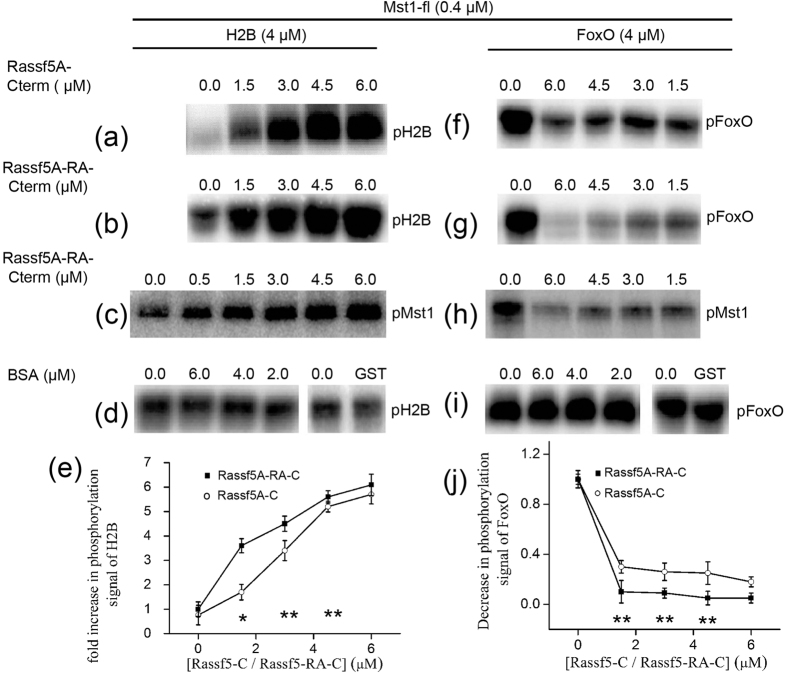
Regulation of kinase activity of Mst1 *in vitro* by Rassf5A constructs. (**a**) and (**f**) SDS-PAGE gel showing the kinase activity of indicated concentrations of purified His_6_-Mst1-fl towards the phosphorylation of its substrates H2B and FoxO in presence of various concentrations of Rassf5A-Cterm; (**b**) and (**g**) Rassf5A-RA-Cterm respectively. (**c**) SDS-PAGE gel depicting the autophosphorylation signal of Mst1-fl upon addition of Rassf5A-RA-Cterm in presence of substrates histone H2B and (h) FoxO respectively. The signal depicted is the incorporation of the γ-^32^P label onto the histone H2B (a,b), FoxO (**f,g**) and Mst1-fl (**c,h**) respectively. (**d**) SDS-PAGE gel depicting the kinase activity of Mst1-fl in presence of standard control proteins BSA and GST towards its substrates histone H2B and (**i**) FoxO respectively. All the reactions were performed by first incubating Mst1 enzyme in kinase buffer with either of substrates histone H2B and FoxO and various concentrations of Rassf5A constructs for 30 minutes in different experiments. The control indicates the absence of either Rassf5A-Cterm or Rassf5A-RA-Cterm in the reaction mixture. (**e**) Graphical representation of fold increase in the phosphorylation signal of histone H2B in the absence and presence of various concentrations of Rassf5A-Cterm and Rassf5A-RA-Cterm. (**j**) Graphical representation of the decrease in phosphorylation signal of FoxO by Mst1 in the absence (control) and presence of Rassf5A-Cterm and Rassf5A-RA-Cterm respectively. Statistical analysis was performed by comparing the phosphorylation of H2B/FoxO at individual concentrations of Rassf5 with control (no Rassf added). P < 0.05 was considered significant (*P < 0.05, **P < 0.001).

**Figure 5:**
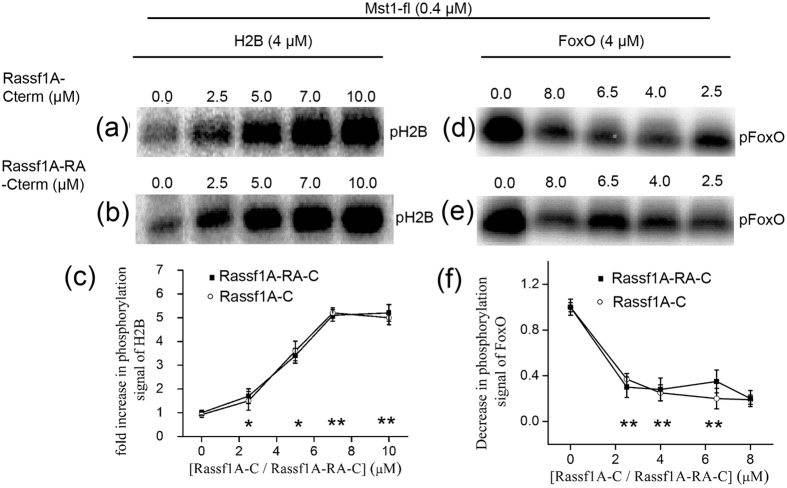
Regulation of kinase activity of Mst1 *in vitro* by Rassf1A constructs. (**a**) and (**d**) SDS PAGE gel showing the kinase activity of indicated concentrations of purified His_6_-Mst1-fl towards the phosphorylation of its substrates H2B and FoxO in presence of indicated concentrations of Rassf1A-Cterm; (**b**) and (**e**) Rassf1A-RA-Cterm respectively. The signal depicted is the incorporation of the γ-^32^P label. The control indicates the absence of either Rassf1A-Cterm or Rassf1A-RA-Cterm in the reaction mixture. (**c**) Graphical representation of fold increase in the phosphorylation signal of histone H2B in the absence and presence of various concentrations of Rassf1A-Cterm and Rassf1A-RA-Cterm. (**d**) Graphical representation of the decrease in phosphorylation signal of FoxO by Mst1 in the absence (control) and presence of Rassf1A-Cterm and Rassf1A-RA-Cterm respectively. Statistical analysis was performed by comparing the phosphorylation of H2B/FoxO at individual concentrations of Rassf1 with control (no Rassf added). P < 0.05 was considered significant (*P < 0.05, **P < 0.001).

After establishing the interaction profile of Mst1 with Rassf1/5 set of adaptors, the next important question addressed was; how does this interaction change downstream signalling ? Therefore, to understand the significance of the protein-protein interactions, *in vitro* kinase assays were designed to quantitatively measure the enhancement or suppression of Mst1 activity towards its two substrates; histone H2B and FoxO. As depicted in Fig. 4a, the addition of Rassf5A-Cterm increases the phosphorylation signal of histone H2B and augmenting the RA domain to Rassf5A-Cterm construct further escalates this signal (Fig. 4b and Supplementary Table S1). Even the autophosphorylation of Mst1 is enhanced in presence of the Rassf1A/5A-RA-Cterm (Fig. 4c). The control experiments with BSA and GST did not show any effect on the kinase activity of Mst1 (Fig. 4d). Identically designed experiments in presence of increasing concentrations of both Rassf1A-Cterm and Rassf1A-RA-Cterm (Fig. 5a,b and Supplementary Table S1) confirmed that the addition of Rassf1A-Cterm exhibited a similar effect to Rassf5A-Cterm. However, in contrast to Rassf5A-RA-Cterm, presence of RA domain to Rassf1A-Cterm does not add to the existing signal, demonstrating that in the case of Rassf1A this domain is not involved in regulation of Mst1 activity (Fig. 5a,b and Supplementary Table S1). From the assay results, it is evident that Rassf proteins positively regulate Mst1-fl activity towards histone H2B. In addition, in the case of Rassf5, the RA domain appears to be responsible for further enhancing this effect. This observation is in consort with our previous findings where a differential binding profile was also observed for Rassf5A-RA-Cterm and Rassf1-RA-Cterm and the SPR results indicated that Rassf5A is the preferred binding partner for Mst1.

In contrast to the enhancement of phosphorylation observed for histone H2B, in the case of FoxO, Mst1-fl mediated phosphorylation results in an opposing effect. As shown in Figs 4f,g, 5d and e, both Rassf5A and Rassf1A significantly inhibit phosphorylation of Mst1-fl towards FoxO. Control experiments with BSA and GST were also performed to confirm that the effects observed were not spurious (Fig. 4i). Our results demonstrated that the addition of Rassf5A-Cterm decreases the phosphorylation signal of FoxO by 60% (Fig. 4f,j and Supplementary Table S1) whereas; the addition of same concentrations of Rassf5A-RA-Cterm decreases the signal by 80% (Fig. 4g,j and Supplementary Table S1). Additionally, the autophosphorylation signal of Mst1 also appears to have decreased (Fig. 4h), signifying that Mst1 is unable to get properly activated. It was again noticed here that in contrast to Rassf5A, the addition of RA domain to Rassf1A-Cterm doesn’t significantly change the activity profile of Mst1 towards FoxO (Fig. 5f and Supplementary Table S1). Although both Rassf1A and Rassf5A are adaptor proteins, the subtle differences in their binding and activity profile, in presence of their RA domains reflects that this domain plays a significant role in differentiation of the adaptors in presence of their cognate proteins. Both the SPR and biochemical experiments indicate that Rassf5A is the primary protein that is involved in the Rassf-Mst1 signalling cascade.

**Figure 6:**
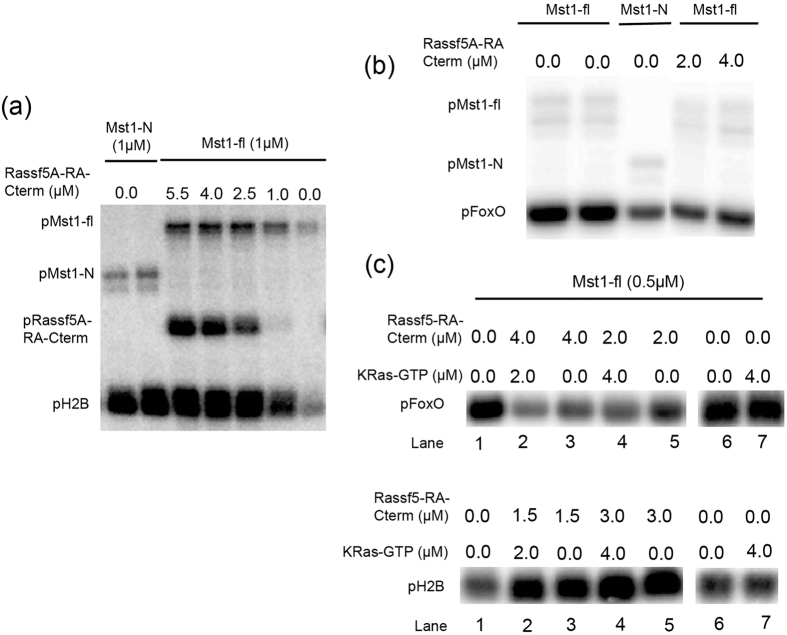
Switching of Mst1-fl as Mst1-N in presence of Rassf proteins. SDS-PAGE gels showing the kinase activity of indicated concentrations of both purified His_6_-Mst1-fl and His_6_-Mst1-N towards the substrate (**a**) H2B and (**b**) FoxO in the absence (control) and presence of various concentrations of Rassf5A-RA-Cterm. In panel B, the concentration of Mst1-fl in lane 1, 2, 4, 5 is 1 μM and in lane 3, the concentration of Mst1-N is 1 μM (**c**) SDS-PAGE gels depicting the activity of Mst1-fl towards its substrates H2B and FoxO in presence of indicated concentrations of Rassf5A-RA-Cterm and GTP-KRas. The signal depicted is the incorporation of the γ-^32^P label.

Further, to confirm the degree of activation of Mst1-fl activity in the presence of Rassf proteins towards histone H2B, the phosphorylation signal was compared to that observed with the cognate form of Mst1: the caspase cleaved form, Mst1-N (Fig. 6a). Using equal amounts of enzymes for equal duration, the two signals were found to be at par. Similarly, the phosphorylation signal of FoxO by Mst1-fl in presence of Rassf proteins was also observed to be equivalent to that observed for Mst1-N (Fig. 6b). These observations confirm that in presence of Rassf5A, full length Mst1 behaves like the caspase cleaved version of the enzyme. The C-terminal of Mst1 engages in interaction with the SARAH and RA domains of Rassf5A, thereby obliterating its regulatory function.

### Effect of Ras on Rassf mediated activity of Mst1-fl

Since Rassf binds both Mst1 and Ras-GTP, there might be a possibility for the formation of the Ras-Rassf-Mst1 ternary complex. Here, we explore if this complex affects the phosphorylation status of both substrates histone H2B and FoxO. Results show that in an *in vitro* setting, GTP-Ras has a subsidiary effect on Mst1 activity towards its substrates (Fig. 6c, lane 6 and 7). It appears to play a marginal role in assisting Rassf in inducing the switch in the activity profile of Mst1 (Fig. 6c, lane 2–5). Statistical analysis on the data in presence of KRas also did not bring out any clear trend. Therefore, we believe that this signalling cascade is likely more complex and further *in vivo* experimentation is required to confirm these findings. One possibility that arises from our study here is that Mst1, Ras-GTP and Rassf5A, may not work in consort. It is also possible that *in vivo* the localization status of Rassf may be altered with the help of other adaptor proteins and depending on its location, an activation event is mediated. Studies have shown that apoptotic activity of Mst1 is enhanced by recruiting it to the membrane^10^,^31^. It is plausible that Ras-GTP assists in function by targeting the Ras-Rassf1A/5A-Mst1 complex to the membrane.

### Mechanistic model for regulation of Mst1 kinase activity by Rassf family

**Figure 7:**
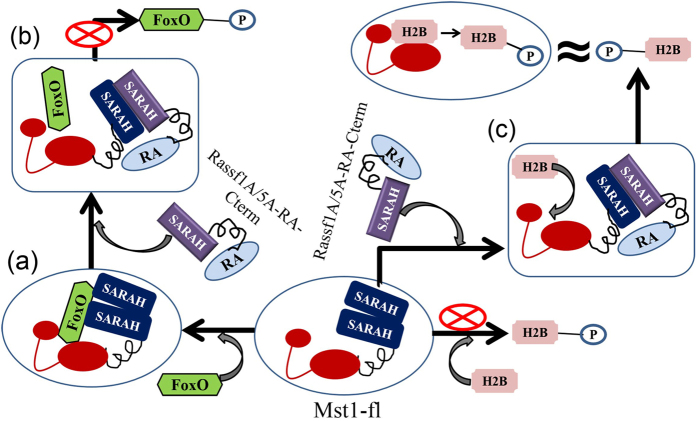
Schematic mechanistic model explaining the mode of regulation of Mst1 kinase activity towards its substrates FoxO and histone H2B by Rassf adaptor proteins. (**a**) The binding site of FoxO requires both kinase and SARAH domain of Mst1. Homo-dimerization of Mst1 via its SARAH domain favours its kinase activity towards FoxO while it inhibits H2B phosphorylation. (**b**) Heterodimerization of Mst1- SARAH with Rassf1A/5- SARAH disrupts the FoxO binding site thereby preventing FoxO phosphorylation. (**c**) The heterodimerization of Mst1- SARAH with Rassf1A/5- SARAH allows the binding of histone H2B in the active site of Mst1 that further promotes its efficient phosphorylation.

Based on the collective experiments performed in this work and previous reports on differential activity of various isoforms of Mst, a mechanistic model for its regulation by Rassf family of proteins has been proposed (Fig. 7). In the absence of Rassf proteins, FoxO is the primary substrate for Mst-fl. Therefore, we propose that efficient phosphorylation of FoxO only occurs when the two SARAH domains of Mst1 are able to interact via formation of a homodimeric complex. It is likely that the binding interface of FoxO, apart from comprising of the usual kinase domain binding cleft also consists of certain SARAH domain residues. Once the DNA binding domain of FoxO is properly positioned via additional anchoring by the SARAH homodimer, efficient turnover is facilitated (Fig. 7a). The addition of Rassf adaptor however, breaks the Mst1 homodimer, resulting in the preferential formation of the SARAH– SARAH heterodimer. This prevents the SARAH domain of Mst1 from adopting a conformation conducive for FoxO phosphorylation, obliterating its activity (Fig. 7b).

Contrastingly, in the case of histone H2B, the caspase cleaved form, Mst1-N, is the primary isoform that efficiently phosphorylates it, whereas, Mst-fl exhibits marginal activity. Therefore, it is likely that the SARAH-SARAH homodimerization of Mst1-fl impedes effective phosphoryation of histone H2B by not allowing it to properly dock into the kinase active site. It is the disruption of the native Mst1-fl homodimer that appears to be the key to Mst mediated serine 14 phosphorylation of histone H2B. During activation of Mst1 via the caspase pathway, this homodimerization domain is automatically cleaved at the ‘DEMD’ sequence yielding Mst1-N. Here, we show that presence of Rassf obliterates the need of cleavage as it enables the sequestration of the inhibitory Mst1 C-terminal SARAH domain via formation of a heterodimeric complex (Fig. 7c). The augmentation of the RA domain of Rassf5A further helps in removing interference caused by RR domain leading to full activation of Mst1-fl towards histone H2B. Therefore, the interaction of the Rassf adaptors with full length Mst1 on one hand triggers histone H2B mediate apoptosis via a caspase independent pathway and on the other hand, obliterates the house keeping activities of the Mst1-FoxO pathway.

Rassf set of tumor suppressors control various processes ranging from cell migration, mitotic cell division, inflammation to modulating apoptosis. In this study the mechanistic model proposed here helps in understanding the signalling cascade that is initiated upon interaction of Rassf with Mst1 kinase. The direct interaction of Rassf with the pro-apoptotic kinase appears to bypass the need of activation of this enzyme via the caspase mediated activation of this enzyme. While phosphorylation of FoxO by Mst1 full length appears to be a housekeeping function to ensure recovery of the cell from small damages, the direct activation by Rassf appears to be a distress signal to prevent tumorigenesis. In conclusion, both the SPR experiments and kinase assays have shown that multiple domains of Rassf and Mst1 play a role in modulating kinase activity of Mst1-fl towards its two substrates histone H2B and FoxO. Interaction of Rassf with Mst1 appears to pass a signal that activates the dormant Mst-fl protein to partake in chromatin remodelling such that apoptosis is initiated. This is the first report which explores this aspect of Rassf function. Activation of Mst-fl via this route seems to bypass the normal caspase cleavage route otherwise undertaken. Therefore, to develop a deeper understanding, the next step is to explore the biological implications of this finding in an *in vivo* setting.

## Methods

### Plasmid construction

The full length human Mst1 (1–487) and the kinase domain of Mst1 (1–334) were sub cloned in the Pachis-Tev baculoviral transfer vector from the clone 7939613 (open biosystems). All Rassf1A constructs (Rassf1A-Cterm, Rassf1A-RA-Cterm) and K-Ras were cloned in pGEX4T3. The Rassf5A constructs (Rassf5A-Cterm, Rassf5A-RA-Cterm) were cloned both in pET vector system and pGEX4T3 system. The Rassf5A constructs expressed in pET vector system were used for radioactive kinase assays whereas Rassf5A constructs expressed in pGEX4T3 system were used to perform SPR experiments. The deletion mutants of Rassf1A and Rassf5A (Rassf1A/5-RA) were made by site directed mutagenesis (SDM) method using Kapa HiFi polymerase enzyme (Quikchange SDM, Stratagene).

### Purification of proteins expressed with baculo virus system

The recombinant Mst1 full length and Mst1-N protein were expressed in Sf9 cells. All procedures on the protein including growth, purification were performed as described previously^17^ and were purified as 6X His-tagged proteins.

### Bacterial expression of Rassf and Mst1 constructs in pET vector system

All the Mst1 constructs used for BIAcore studies, Rassf5A constructs and the deletion mutant of Rassf5A were expressed as 6X-His tag fusion protein to facilitate purification. The protein was expressed in *E.coli* BL21 (DE3) cells and was purified using Qiagen Ni-NTA resin. The bound protein was eluted with 100 mM imidazole. The protein was further purified by size exclusion chromatography using Superdex 75 column in 20 mM HEPES (pH 7.5), 100 mM NaCl. The protein fractions from the column were pooled together and concentrated, flash frozen in liquid N_2_ and stored at −80°C until use.

### Bacterial expression of Rassf constructs in pGEX4T3 vector system

The mouse constructs of Rassf1A, Rassf5A and K-Ras that were cloned in pGEX4T3 vector system are expressed as GST fused proteins. The GST fusion protein was purified by sonication of cell pellet in ice cold STE Buffer (10 mM Tris-HCl, pH 8.0, 1 mM EDTA, 150 mM NaCl) followed by washing with ice-cold Phosphate buffer saline (1X PBS) (137 mM NaCl, 2.7 mM KCl, 10 mM Na_2_HPO_4_, 2 mM KH_2_PO_4_). The GST fusion protein was eluted with elution buffer (20 mM Tris pH 8.0, 15 mM reduced glutathione). Reduced glutathione from the elute was removed by dialyzing against 1X PBS. The proteins were further purified by using Superdex 75 and Superdex 200 columns in PBS buffer and made into aliquots for further storage.

### H2B and FoxO

Histone H2B was purchased from New England Biolabs (NEB). The DNA binding domain (DBD) of FoxO (AA 151-266) was cloned in pETDuet-1 vector containing the gene for yeast SMT3, an ubiquitin-like protein of the SUMO family. All procedures on the FoxO protein including growth and purification were performed as described previously^17^.

### Kinase Assay

The protein kinase assay was performed by monitoring the incorporation of radiolabelled *γ*^32^P into the substrates histone H2B and FoxO. The initial reaction was performed by first incubating Mst1 enzyme in kinase buffer (50 mM Tris pH 7.5, 0.1 mM MgCl_2_, 0.1 mM DTT) with substrates histone H2B and FoxO at a final concentration of 4 μM and various concentrations of Rassf1A or Rassf5A constructs for 30 min. The reaction was started by adding 2 *μ*Ci of labelled *γ*-^32^P ATP, 100 μM ATP was used in each reaction. After 30 min incubation, the reaction was stopped by adding 6 μl of 10X SDS loading dye and the samples were boiled for 5 min at 100 °C. 20 μl of the reaction volume was loaded in each well of SDS gel. The gel was transferred to the cassette for 1 h exposure and signal was measured using a phosphoimager. The intensity of each phosphorylation signal was calculated by image quant software from GE Health care and the graphs were plotted using Origin 8.0 software. For the kinase assays using K-Ras, the purified K-Ras protein was loaded with GTP at 4 °C using the protocol described previously^32^ to get activated K-Ras. Later equal concentrations of the activated K-Ras and Rassf5A-RA-Cterm were incubated for half an hour before the Mst1 kinase assay was performed. The remaining kinase assay procedure is same as described above. All the kinase assay experiments were performed in triplicates. Statistical analysis was performed by comparing the phosphorylation of H2B/FoxO at all the concentrations of Rassf with control (absence of Rassf proteins). The data were analyzed by using the unpaired Student’s t test in GraphPad Prism software and the data represent the mean ± SEM. P < 0.05 was considered significant. (*P < 0.05, **P < 0.001) (Figs 4e,j, 5c,f and Supplementary Figure 4).

### SPR characterization - Immobilization of anti GST antibody and ligand capture

Recombinant proteins fused with GST are immobilized on a sensor chip surface through the GST/anti-GST antibody using GST capture kit from GE Health care. Later the ligand capture was carried out by binding both GST - Rassf fusion protein and the GST protein alone to the immobilized anti GST antibody on two different channels at a flow rate of 10 μl/min, for 180 s with stabilization period of 30 s. Both of these proteins were diluted in HBS-N running buffer to get the final concentration of 10–30 μg/ml (the final concentration for Rassf1A constructs are 30 μg/ml, whereas for Rassf5A constructs is 10 μg/ml). The channel containing GST tagged - Rassf fusion protein was used as the active surface and the channel that contains only GST protein without any fusion partner was used as reference surface. Regeneration is accomplished by injecting a glycine-HCl solution at pH 2.2 over the sensor chip surface. In this approach, the antibody serves as a universal adaptor, permitting the capture of all GST-fusion proteins on the sensor chip and any non-specific binding to GST protein will get invalidated by subtracting the response of reference from the active surface.

### SPR Kinetic measurements

All the Mst1 constructs acting as analytes were diluted in running buffer (0.1 M HEPES, 1.5 M NaCl) to final concentrations of 50, 25, 12.5, 6.25, 3.125, 1.56 and 0.7 μM. Each concentration was injected over immobilized peptides in both active and reference channels during the association phase for 300 s at a flow rate of 30–45 μl/min along with the running buffer HBS-N. Later the same running buffer was then flushed for 600–900 s at a flow rate of 30 μl/min to initiate the dissociation phase. The biosensor surface regeneration was carried out by injecting 10 mM Gly-HCl solution pH 2.1 for 60 s at a flow rate of 30 μl/min. One middle concentration of each sample was injected in duplicate in at least two separate experiments. The interaction of Mst1 constructs with immobilized Rassf constructs was monitored in real time and expressed with a sensorgram reporting response in relative units. All binding curves were corrected for background and bulk refractive index contribution by subtraction of the reference flow cells response from active surface. Models were fitted locally across the data sets and for a single concentration. Obtained kinetic data were analyzed using the Biacore T200 Evaluation software 2.0 (GE Healthcare) under a theoretical 1:1 interaction kinetic model describing 1:1 binding between analyte (*A*) and ligand (*B*) in order to calculate the association rate (*k*_a_) and the dissociation rate (*k*_d_) and the equilibrium dissociation constant (*K*_D_ = *k*_d_/*k*_a_) by non-linear fitting. The chi^2^ values for

sensorgram curve fits were equal to or less than 15 for all curves.

### Circular Dichroism

Far-UV CD spectra (200–250 nm) of Rassf1A-Cterm (0.4 mg/ml), Rassf1A-RA-Cterm (1.2 mg/ml), Rassf5A-Cterm (0.5 mg/ml), Rassf5A-RA-Cterm (0.4 mg/ml), Mst1-fl (0.8 mg/ml), Mst1-RR-C (1.2 mg/ml) and Mst1-N (0.7 mg/ml) in 1X PBS were recorded on Jasco 815 CD spectrophotometer using a 1 mm quartz cuvette. Scans were accumulated with a scan rate of 50 nm/min and bandwidth of 1 nm.

## Additional Information

**How to cite this article:** Bitra, A. *et al*. Rassf Proteins as Modulators of Mst1 Kinase Activity. *Sci. Rep.* **7**, 45020; doi: 10.1038/srep45020 (2017).

**Publisher's note:** Springer Nature remains neutral with regard to jurisdictional claims in published maps and institutional affiliations.

## Supplementary Material

Supplementary Information

## Figures and Tables

**Table 1:** Kinetic parameters of various constructs of Mst1 interaction with Rassf1A/5.

Ligand	Analyte	*k*_a_ × 10^−3^ Ms	*k*_d_ × 10^4^ s	*K*_D_ × 10^6^ M^−1^	Std error
Rassf1A-Cterm	Mst1-C	0.32–0.33	1.85–2.81	0.87	±0.2
Rassf5A-Cterm	Mst1-C	0.20–0.32	0.81–0.97	0.31	±0.15
Rassf1A-Cterm	Mst1-RR-C	0.17–0.43	4.22–9.61	2.33	±0.16
Rassf5A-Cterm	Mst1-RR-C	0.11–0.19	1.52–1.78	1.04	±0.22
Rassf1A-RA-Cterm	Mst1-C	0.13–0.16	4.34–5.12	3.13	±0.17
Rassf5A-RA-Cterm	Mst1-C	0.21–0.35	0.31–1.53	0.29	±0.19
Rassf1A-RA-Cterm	Mst1-RR-C	0.12–0.16	4.31–5.05	3.27	±0.36
Rassf5A-RA-Cterm	Mst1-RR-C	0.17–0.27	0.66–1.18	0.41	±0.04
Rassf5A-RA	Mst1-RR-C	0.38–0.53	3.13–3.16	0.81	±0.15
Rassf5A-RA	Mst1-C	No binding			
Rassf5A-Cterm	Mst1-fl	0.11–0.13	0.03–0.04	0.035	±0.005

Association rates (*k*_a_), dissociation rates (*k*_d_), and dissociation constants (*K*_D_ = *k*_d_/*k*_a_), standard deviation in the measured *K*_D_ values are given.
