# Supplementary material for: Rassf Proteins as Modulators of Mst1 Kinase Activity
Source: Sci Rep. 2017 Mar 22;7:45020. doi: 10.1038/srep45020 (PMC5361201; doi:10.1038/srep45020)
Supplement: Supplementary Information [file srep45020-s1.pdf]

SUPPORTING INFORMATION

Rassf Proteins as Modulators of Mst1 Kinase Activity

Aruna Bitra[1], Srinivas Sistla[2], Jessy Mariam[1], Harshada Malvi[1], and Ruchi Anand[1*]

[1] Department of Chemistry, Indian Institute of Technology Bombay, Powai, Mumbai, India 400076

[2] GE Healthcare Life Sciences, John F Welch Technology Centre, Whitefield Road, Bangalore,
India 560048

*To whom correspondence should be addressed: Ruchi Anand, Department of Chemistry, Indian Institute of Technology Bombay, Powai, Mumbai, India. Tel.: (022) 2576-7165; Fax: (022) 2576-7152; E-mail: ruchi@chem.iitb.ac.in

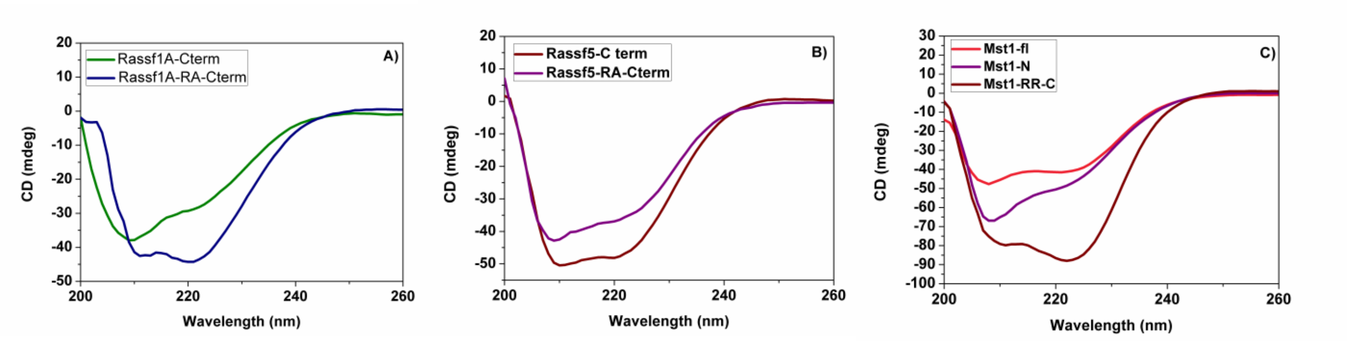

Supplementary figure S1: Far-UV CD spectra of A) Rassf1A-Cterm (0.4 mg/ml) and Rassf1A-RA-Cterm (1.2 mg/ml) B) Rassf5-Cterm (0.5 mg/ml) and Rassf5-RA-Cterm (0.4 mg/ml) and C) Mst1-fl (0.8 mg/ml), Mst1-N (0.7 mg/ml) and Mst1-RR-C (1.2 mg/ml) in 1X PBS

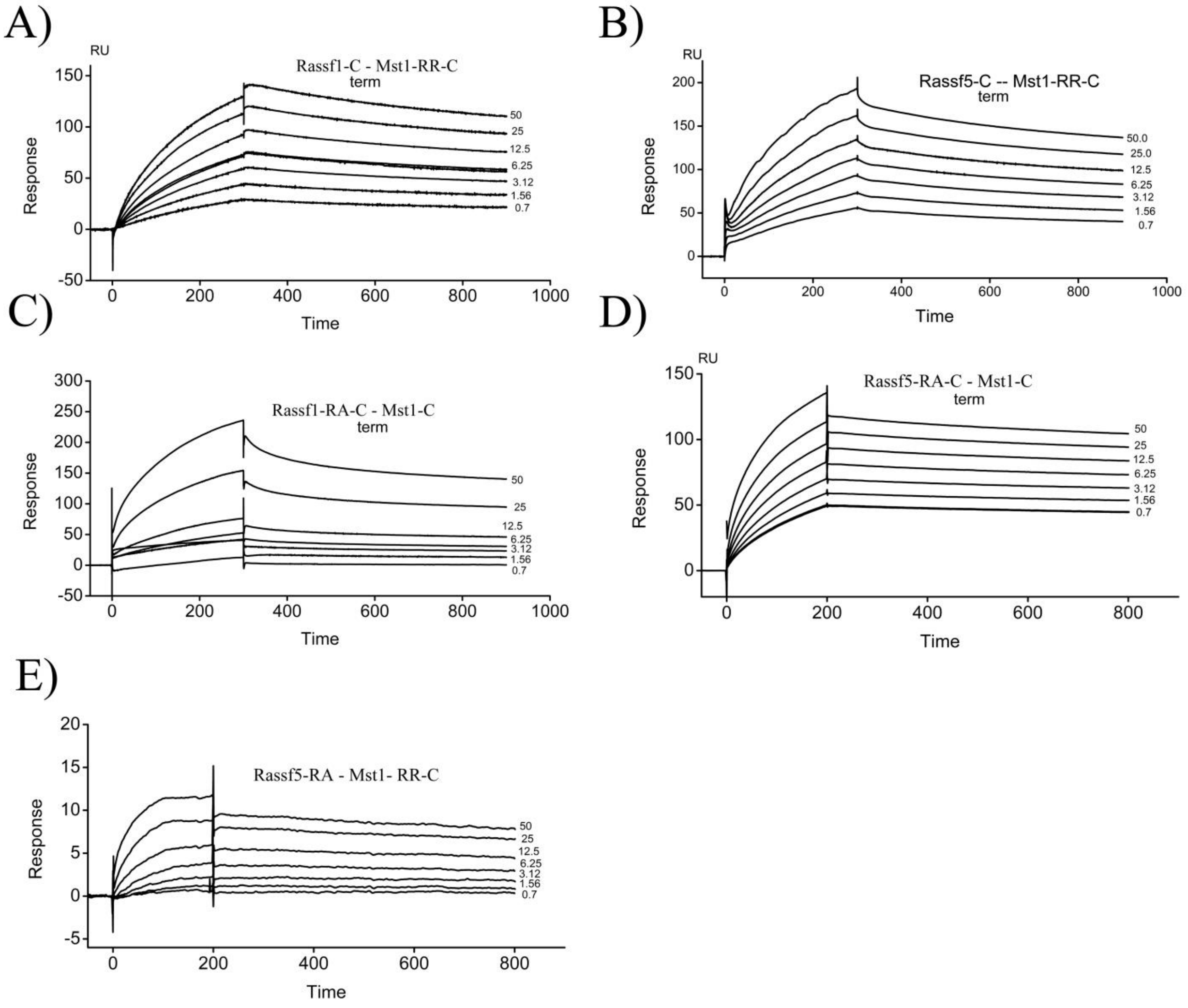

Supplementary figure S2: BIAcore analysis for the interaction of Mst1-C and Mst1-RR-C with Rassf1/5-Cterm and Rassf1/5-RA-Cterm surfaces. Sensorgrams are presented for the interaction of Mst1-RR-C with (A) Rassf1A-Cterm (B) Rassf5-Cterm; interaction of Mst1-C with (C) Rassf1A-RA-Cterm (D) Rassf5-RA-Cterm respectively. (E) SPR data for the interaction of Rassf5-RA with Mst1-RR-C. A concentration range (50, 25, 12.5, 6.25, 3.125 and 0.7 µM) of Mst1 analytes was analyzed on Rassf1A/5-Cterm and Rassf1A/5-RA-Cterm biosensor surfaces.

```
Rassf1A-Cterm  290   EVNWDAFSMPELHNFLRILQREEEEHLRQILQKYSRCRQKIQEALHACP--LG 51
Rassf5-Cterm  361    EVEWDAFSIPELQNFLTILEKEEQDKIHQLQKKYNKFRQKLEEALRESQGKPG 53
                     **:*****:***:*** **::**:::::*: :**.: ***::***: .    *
```

Supplementary figure S3: (a) Sequence alignment of SARAH domains of both Rassf1A and Rassf5. The residues conserved in both the proteins involved in interaction with Mst1 SARAH domain are colored red. The residues in Rassf1A-Cterm that were differing from Rassf5-Cterm interacting residues with Mst1-C were colored green.

.

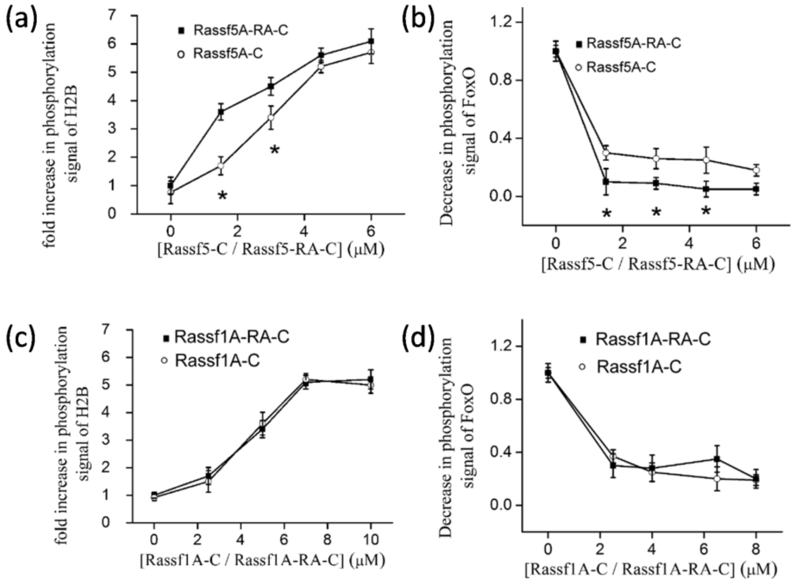

Supplementary figure S4: Differential phosphorylation by Mst1 in presence of the RA domain augmented Rassf5. Graphs a) and c) show effect on histone H2B phosphorylation b) and d) on FoxO phosphorylation status. * represents P < 0.05. Comparison is between RA augmented version of Rassf1/5-C and Rassf1/5-RA-C.

Statistical analysis was performed with GraphPad Prism software by considering the phosphorylation siganl of H2B/FoxO in presence of Rassf5A-Cterm and Rassf5-RA-Cterm as group A and group B respectively and then calculated the significant difference between them. Wherever appropriate, data were analysed by using the Student's t test and unless otherwise indicated, data represent the mean ± SEM. **P*<0.05 was considered significant. The statistical analysis depicts significant difference between Rassf5-Cterm and Rassf5-RA-Cterm (at concentrations 1.5µM and 3 µM) towards Mst mediated H2B/FoxO phosphorylation. No significant difference was observed between Rassf1A-Cterm and Rassf1A-RA-Cterm towards Mst mediated H2B/FoxO phosphorylation.

Supplementary Table 1: Quantitative measurements depicting the regulation of kinase activity of Mst1 towards its substrates in presence of Rassf proteins. The percentage phosphorylation of H2B and FoxO by Mst1-fl in the control (absence of either BSA or Rassf1A/5) in each set of experiment is considered as 100%.

	% phosphorylation of histone H2B by Mst1-fl	% phosphorylation of FoxO by Mst1-fl
Rassf5-Cterm (1.5 μM)	174 ± 32	36 ± 8
Rassf5-RA-Cterm (1.5 μM)	360 ± 29	16 ± 4
Rassf1A-Cterm (2.5 μM)	159 ± 39	45 ± 14
Rassf1A-RA-Cterm (2.5 μM)	172 ± 31	41 ± 9
BSA (μM) as standard control	87 ± 2	94 ± 3
